# The association between AI dependence, AI literacy, and critical thinking ability among nursing interns: a multicenter cross-sectional study

**DOI:** 10.3389/fpsyg.2026.1823047

**Published:** 2026-04-15

**Authors:** Wei Cui, Xiaping Zhang

**Affiliations:** 1 Clinical Nursing Teaching and Research Section, The Second Xiangya Hospital, Central South University, Changsha, Hunan, China; 2 Department of Geriatrics, The Second Xiangya Hospital, Central South University, Changsha, Hunan, China; 3 Department of Vascular Surgery, The Second Xiangya Hospital, Central South University, Changsha, Hunan, China

**Keywords:** artificial intelligence, critical thinking ability, mediation analysis, nurse education, nursing interns

## Abstract

**Aim:**

To examine the associations between Artificial intelligence (AI) dependence and critical thinking ability among nursing interns and to explore the potential mediating role of AI literacy.

**Methods:**

A convenience sampling method was used to select nursing interns recruited from multiple hospitals in Hunan, China between January and February 2026. A general information questionnaire, AI literacy scale, AI dependence scale, and critical thinking ability scale were used to conduct the online questionnaire survey. Data were analyzed using descriptive statistics, Pearson correlation analysis, and regression-based mediation analysis (PROCESS Model 4) with 5,000 bootstrap resamples.

**Results:**

A total of 517 nursing interns were included in the final analysis. AI dependence was positively associated with AI literacy and critical thinking ability (all *p* < 0.001). After adjusting for covariates, AI literacy partially mediated the relationship between AI dependence and critical thinking ability. The indirect effect was significant [95%CI (0.399, 0.610)], and the model explained 52.0% of the variance in critical thinking ability.

**Conclusion:**

AI dependence was positively associated with critical thinking ability among nursing interns, both directly and indirectly through AI literacy. These findings suggest that integrating AI literacy training into nursing education may help optimize the educational benefits of AI.

## 1 Introduction

Artificial intelligence (AI) has rapidly become embedded in higher education, transforming how students access information, organize knowledge, and accomplish academic tasks (Lee, 2024; Boscardin et al., 2024). Generative AI systems, especially large language models, can quickly provide structured explanations of users’ questions (Reddy, 2024). In nursing education, AI-powered tools are now widely adopted across various teaching and learning contexts (El Arab et al., 2025; Castonguay et al., 2023). These technologies are used across a range of educational activities, including nursing simulation education (Chan et al., 2025), academic writing (Tseng et al., 2025), and clinical case analysis (Akutay et al., 2024).

However, generative AI systems have inherent limitations. Large language models may occasionally produce inaccurate or misleading information (Messeri and Crockett, 2024). As these systems become more embedded in nursing education, it is important to consider how students interpret and rely on such outputs. In particular, educators question whether sustained reliance on AI tools may shape the development of critical thinking ability, a core competency that underpins clinical judgment, evidence-based decision-making, and patient safety in nursing practice (Castonguay et al., 2023).

Critical thinking ability is defined as the ability to apply higher-order cognitive skills, including conceptualization, analysis, evaluation, and reflection (Papp et al., 2014). It plays a central role in nursing practice, as it underlies clinical judgment, problem-solving, and evidence-based decision-making (Yue et al., 2017). AI dependence refers to the extent to which individuals rely on artificial intelligence systems during learning and problem-solving processes (Robayo-Pinzon et al., 2025). In this study, AI dependence primarily reflects patterns of engagement with AI tools, encompassing both functional dependence and emotional dependence. Functional dependence refers to reliance on AI for task-related activities, whereas emotional dependence reflects a psychological tendency to trust or prefer AI-assisted outputs. Concerns have been raised that uncritical reliance on AI may be associated with diminished higher-order cognitive engagement (Alhur et al., 2025). However, Cognitive Load Theory (CLT) offers a more nuanced perspective (Zou et al., 2025). According to CLT, learners have limited working memory capacity. Learning is most effective when cognitive resources are used efficiently (Szulewski et al., 2021). Educational tools that reduce extraneous load and support germane load may facilitate higher-order thinking (Zou et al., 2025). AI tools may reduce extraneous cognitive load by organizing information, summarizing materials, and providing structured explanations. When routine or time-consuming tasks are supported by AI, students may have more cognitive resources available for deeper analysis and evaluation. In this sense, AI dependence does not necessarily undermine thinking; rather, it may be associated with more efficient allocation of cognitive resources.

AI literacy refers to the knowledge and skills that enable students to understand, evaluate, and use artificial intelligence tools effectively in academic tasks (Simms, 2025). It includes awareness of how AI systems work, recognition of their limitations and possible biases, and the ability to assess the accuracy and logic of AI-generated information (Simms, 2025). Since AI literacy involves the ability to critically evaluate and appropriately use AI systems, it may help explain how AI dependence is associated with critical thinking ability. Students with higher AI literacy are more likely to question AI outputs, verify information, and integrate AI-generated content into their own analysis (El-Sayed et al., 2025). According to CLT, AI literacy helps students manage their cognitive resources. It can reduce unnecessary cognitive load while maintaining meaningful cognitive effort through active evaluation and reflection. In addition, repeated interaction with AI tools may provide opportunities for learning, exploration, and feedback, thereby facilitating the development of AI literacy through use and experience. Therefore, AI literacy may represent a potential explanatory pathway in the relationship between AI dependence and critical thinking ability. Although greater AI dependence may increase students’ interaction with AI systems, the extent to which such engagement is associated with deeper reasoning or more superficial processing may depend on students’ level of AI literacy.

Despite the theoretical relevance of these constructs, empirical research examining the association between AI dependence and critical thinking remains limited. Existing studies have largely focused on AI readiness (Labrague et al., 2023), attitudes (Labrague et al., 2023), and integrity concerns (Francis et al., 2024). These studies provide valuable descriptive insights but offer limited understanding of how AI engagement influences higher-order cognitive processes. It remains unclear whether AI primarily supports analytical reasoning as a cognitive aid or gradually replaces independent thinking as a cognitive substitute in nursing education. In particular, few investigations have examined AI literacy as a potential cognitive-regulatory mediator that may explain how AI dependence is association with critical thinking ability.

Based on this theoretical integration, the following hypotheses were proposed:

*H1*: AI dependence is significantly associated with critical thinking ability among nursing interns.

*H2*: AI dependence is significantly associated with AI literacy.

*H3*: AI literacy is positively associated with critical thinking ability.

*H4*: AI literacy is involved in the association between AI dependence and critical thinking ability, consistent with a mediation model.

## 2 Methods

### 2.1 Design and setting

A multicenter cross-sectional study was conducted among nursing interns from several hospitals in Hunan, China.

### 2.2 Participants

Participants were nursing interns recruited from hospitals. All participants were undertaking clinical internship training at the time of data collection. In the present study, the participants refer to students in clinical internship training, regardless of whether they were enrolled in junior college, bachelor’s, or master’s programs. In the Chinese nursing education context, students from different educational pathways may all enter clinical internship stages prior to graduation. Therefore, participants were classified as nursing interns based on their internship status rather than their degree level. Students who submitted incomplete or invalid questionnaires were excluded from the final analysis. The questionnaire link was distributed through internship coordinators at each hospital. Sample size was calculated using G*Power 3.1.9.7 software (Faul et al., 2007). Given that AI literacy and AI dependence are relatively new constructs in nursing education and prior effect sizes suitable for mediation-based power estimation are limited, a conservative small effect size was assumed (*f*^2^ = 0.02) (Cohen, 1988). With *α* = 0.05, power = 0.80, and two predictors in a multiple regression framework, the minimum required sample size was 485. Considering 10% of potential invalid responses, the recruitment sample size is derived as 539.

### 2.3 Measures

All instruments were administered in Chinese. The original scales were developed and validated in Chinese populations.

#### 2.3.1 General information questionnaire

The variables included gender, age, education, level of internship institution, household, only child, total average monthly household income, AI-related training, and the frequency of AI usage. These variables were selected based on prior literature and their potential influence on AI use, learning experiences, and cognitive outcomes among nursing students.

#### 2.3.2 AI dependence

AI dependence was measured using the Artificial Intelligence Dependence Questionnaire developed by Sun and Bi (2025). The scale comprises 12 items across two dimensions: functional dependence and emotional dependence. Each item was scored on a Likert 5 point scale ranging from 1(very inconsistent) to 5(very consistent). Higher scores indicate greater levels of AI dependence. In the present study, the Cronbach’s *α* coefficient in this study was 0.936.

#### 2.3.3 AI literacy

AI literacy was measured using the AI Literacy Scale developed by Zhou et al. (2024). The scale includes 25 items across four dimensions: AI knowledge, AI skills, AI attitudes and values, and AI ethics. Each item is rated on a Likert 7-point scale ranging from 1 (strongly disagree) to 7 (strongly agree). Higher total scores indicate higher levels of AI literacy. In the present study, the Cronbach’s *α* coefficient for the total scale was 0.969.

#### 2.3.4 Critical thinking ability

Critical thinking ability was measured using the Critical Thinking Scale developed by Hou et al. (2022). The scale comprises 17 items across three dimensions: analytic ability, open-mindedness to criticism, and effort to use critical thinking. Items in the second dimension are reverse scored. Each item is rated on a Likert 7-point scale ranging from 1 (strongly disagree) to 7 (strongly agree). Higher total scores indicate stronger critical thinking ability. In the present study, the Cronbach’s *α* coefficient for the total scale was 0.889.

### 2.4 Data collection

Data were collected using an anonymous web-based questionnaire hosted on Wenjuanxing^1^ between January to February 2026. The survey link was distributed through internship coordinators at participating hospitals. Before accessing the questionnaire, participants were informed about the purpose of the study and provided electronic informed consent. To ensure data quality, responses with missing data or obvious response patterns (e.g., identical answers to all items) were excluded. To reduce the risk of duplicate or invalid responses, the survey platform restricted repeated submissions from the same device/IP address.

### 2.5 Statistical analysis

Data were analyzed using IBM SPSS 27.0 software (IBM, 2020). Descriptive statistics were used to summarize participants’ demographic characteristics and study variables. Continuous variables were reported as means and standard deviations, and categorical variables were presented as frequencies and percentages. Pearson correlation analysis was conducted to examine the relationships among AI dependence, AI literacy, and critical thinking ability. To test the mediating role of AI literacy, a regression-based mediation analysis was performed using the PROCESS macro (Model 4). Bootstrap sampling with 5,000 resamples was applied to estimate the indirect effect and its 95% confidence interval (CI). Before conducting the mediation analysis, regression assumptions were assessed. Linearity and homoscedasticity were examined using residual plots, independence of errors was evaluated using the Durbin–Watson statistic, and influential observations were screened using standardized residuals and Cook’s distance. No serious violations of regression assumptions were identified. In addition, heteroscedasticity-consistent standard errors (HC3) were applied to improve the robustness of the estimates. Multicollinearity among independent variables was assessed using variance inflation factors (VIF) and tolerance values. Harman’s single-factor test was conducted to assess the potential risk of common method bias. Effect sizes were interpreted using model explanatory power (*R*^2^), standardized regression coefficients, and the completely standardized indirect effect. An indirect effect was considered statistically significant if the confidence interval did not include zero. *p* < 0.05 was considered statistically significant.

### 2.6 Ethics statement

This study was approved by the Institutional Review Board of the Second Xiangya Hospital of Central South University (LYEC2026-K0033). All participants provided written informed consent before participation. The study was conducted in accordance with the Declaration of Helsinki.

## 3 Results

### 3.1 Demographic characteristics

**Figure 1:**
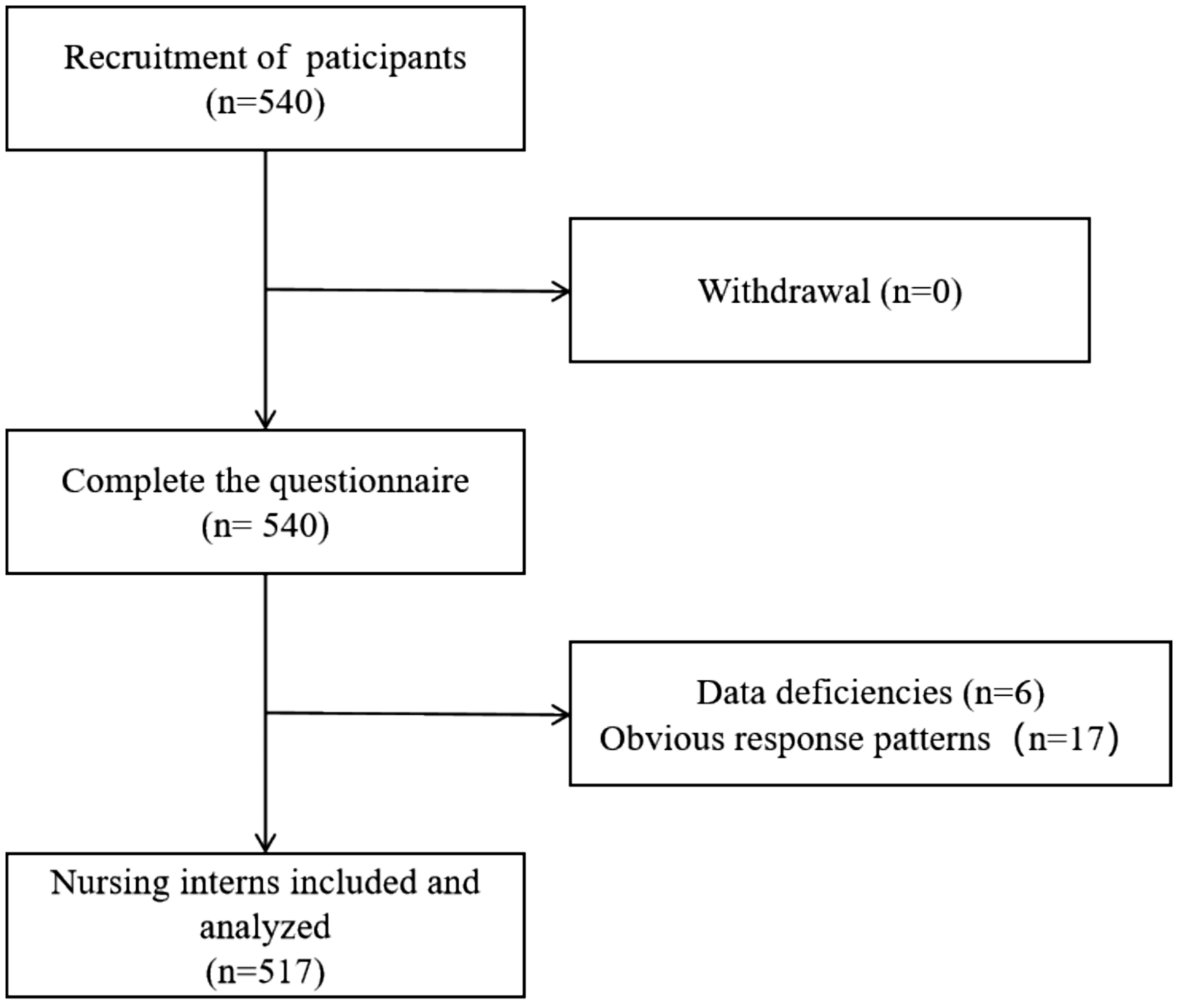
Flow diagram of participant recruitment and inclusion.

A total of 540 questionnaires were collected, and 517 valid responses were included in the analysis. The recruitment flowchart is shown in Figure 1.

Table 1 presented the demographic characteristics of the participants. The sample consisted predominantly of female students (85.5%). Approximately half were undergraduate students (50.1%), and most were from rural areas (72.9%). The majority had received AI-related training (69.6%), and nearly half reported using AI on a weekly basis (46.6%).

**Table 1:** General information of nursing interns (*N* = 517).

Variables	Categories	*N* (%)/Mean ± SD
Gender	Male	75 (14.5)
	Female	442 (85.5)
Age	–	21.07 ± 1.46
Education	Junior college	246 (47.6)
	Bachelor	259 (50.1)
	Master	12 (2.3)
Level of internship institution	Tertiary grade A general hospital	398 (77.0)
	Non-tertiary grade A general hospital	119 (23.0)
Household	City	140 (27.1)
	Countryside	377 (72.9)
Only child	Yes	88 (17.0)
	No	429 (83.0)
Total average monthly household income	<5,000 yuan	230 (44.5)
	5,000 ~ 10,000 yuan	218 (42.2)
	>10,000 yuan	69 (13.3)
AI-related training	Yes	360 (69.6)
	No	157 (30.4)
The frequency of AI usage	Everyday	115 (22.2)
	Weekly	241 (46.6)
	Monthly	114 (22.1)
	Not used in the last 3 months	47 (9.1)

### 3.2 Scores of AI dependence, AI literacy and critical thinking ability of nursing interns

Table 2 summarized the descriptive statistics of the main variables. The mean total scores were (126.75 ± 25.47) for AI literacy, (40.23 ± 9.02) for AI dependence, and (78.60 ± 12.83) for critical thinking ability.

**Table 2:** Descriptive statistics of AI dependence, AI literacy, and critical thinking ability (*N* = 517).

Dimension	Number of entries	Min-Max	Dimension score
AI literacy	25	28 ~ 175	126.75 ± 25.47
AI knowledge	3	3 ~ 21	15.24 ± 3.85
AI skills	8	9 ~ 56	40.58 ± 8.90
AI attitudes and values	6	7 ~ 42	30.04 ± 6.57
AI ethics	8	8 ~ 56	40.90 ± 9.16
AI dependence	12	13 ~ 60	40.23 ± 9.02
Functional dependence	6	6 ~ 30	21.07 ± 4.36
Emotional dependence	6	6 ~ 30	19.17 ± 5.78
Critical thinking ability	17	23 ~ 113	78.60 ± 12.83
Analytic ability	8	10 ~ 56	39.68 ± 8.26
Open-mindedness to criticism	5	7 ~ 33	19.55 ± 3.80
Effort to use critical thinking	4	4 ~ 28	19.37 ± 4.36

### 3.3 Correlation of AI dependence, AI literacy and critical thinking ability of nursing interns

As shown in Table 3, higher levels of AI dependence and AI literacy are related to stronger critical thinking ability among nursing interns. AI dependence was significantly positively correlated with AI literacy (*r* = 0.691, *p* < 0.001) and critical thinking ability (*r* = 0.601, *p* < 0.001). In addition, AI literacy was significantly positively associated with critical thinking ability (*r* = 0.680, *p* < 0.001).

**Table 3:** The correlation of AI dependence, AI literacy and critical thinking ability of nursing interns (*N* = 517).

Variable	AI literacy	AI dependence	Critical thinking ability
AI literacy	1		
AI dependence	0.691^***^	1	
Critical thinking ability	0.680^***^	0.601^***^	1

^***^p<0.001.

### 3.4 Common method bias test

The first unrotated factor accounted for 44.91% of the total variance, which did not exceed the commonly recommended threshold of 50%, suggesting that common method bias was not a serious concern in this study (Podsakoff and Organ, 1986).

### 3.5 Mediation effect of AI literacy between AI dependence and critical thinking ability of nursing interns

A mediation model was tested with AI dependence as the independent variable, AI literacy as the mediator, and critical thinking ability as the dependent variable, while controlling for gender, education level, level of internship institution, household registration, only-child status, family income, AI-related training, and frequency of AI use. Regression diagnostics indicated no serious violations of model assumptions. Multicollinearity was not a concern (tolerance = 0.522, VIF = 1.916), and heteroscedasticity-consistent standard errors (HC3) were applied to ensure robust estimation.

**Figure 2:**
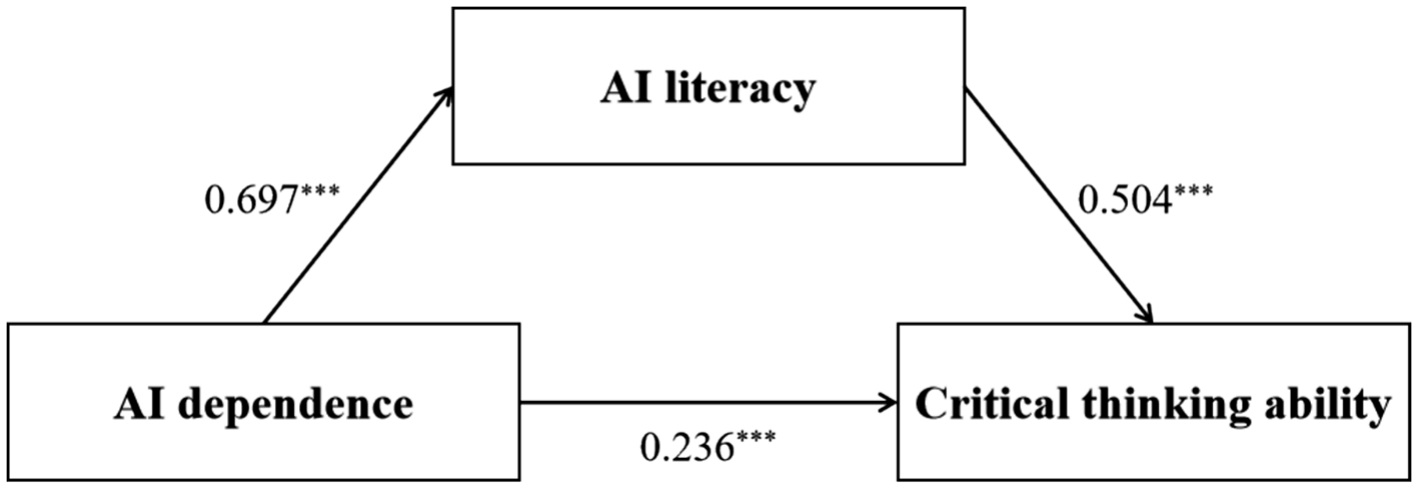
Mediation effect model. Values represent standardized regression coefficients (*β*). ^***^p < 0.001.

AI dependence was significantly positively associated with AI literacy (*B* = 1.967, SE = 0.104, *p* < 0.001), and AI literacy was significantly positively associated with critical thinking ability (*B* = 0.254, SE = 0.025, *p* < 0.001). After including the mediator, AI dependence remained significantly associated with critical thinking ability (direct effect: *B* = 0.335, SE = 0.065, *p* < 0.001), indicating a partial mediation pattern. The total effect of AI dependence on critical thinking ability was also significant (*B* = 0.835, SE = 0.070, *p* < 0.001). Bootstrap analysis with 5,000 resamples showed that the indirect effect of AI dependence on critical thinking ability through AI literacy was significant [indirect effect = 0.500, BootSE = 0.055, 95% CI (0.399, 0.610)]. Because the confidence interval did not include zero, the mediating role of AI literacy was supported. The model explained 52.0% of the variance in critical thinking ability (*R*^2^ = 0.520). The completely standardized indirect effect was 0.352 [95% bootstrap CI (0.285, 0.420)], indicating a moderate mediation effect size. Detailed regression coefficients and bootstrap estimates are presented in Table 4, and the standardized mediation model is illustrated in Figure 2.

**Table 4:** Mediation analysis of AI literacy in the relationship between AI dependence and critical thinking ability (*N* = 517).

Variables	Model path	B	SE/Boot SE	95% CI		*p* values
				LLCI	ULCI	
Path a	AI dependence→AI literacy	1.967	0.104	1.762	2.172	<0.001
Path b	AI literacy→Critical thinking ability	0.254	0.025	0.205	0.304	<0.001
Direct effect (path c’)	AI dependence→Critical thinking ability	0.335	0.065	0.206	0.463	<0.001
Total effect (path c)	AI dependence → Critical thinking	0.835	0.070	0.698	0.971	<0.001
Indirect effect (a × b)	AI dependence→AI literacy→Critical thinking ability	0.500	0.055^*^	0.399	0.610	–

Bootstrap confidence intervals were based on 5,000 resamples. HC3 heteroscedasticity-consistent standard errors were applied. *Boot SE. An indirect effect was considered significant if the 95% bootstrap confidence interval did not include zero.

## 4 Discussion

This study examined the relationships among AI dependence, AI literacy, and critical thinking ability among nursing interns and also assessed their overall levels. The results showed that nursing interns reported moderate levels of AI dependence (40.23 ± 9.02). Functional dependence was slightly higher than emotional dependence, implying that AI was primarily used as a practical support tool rather than as a source of psychological reliance. AI literacy was relatively moderate (126.75 ± 25.47), this finding aligns with previous research by Jiang et al. (2025). Such consistency may reflect the growing normalization of AI technologies within nursing education. Critical thinking ability was at a moderate level (78.60 ± 12.83), which was higher than that reported in a previous study (Nemati-Vakilabad et al., 2023). In that study, students from all academic years were included, including those with limited clinical experience. In contrast, the present study focused only on final-year nursing interns who had already completed a period of clinical training. Greater clinical exposure may be associated with stronger analytical reasoning and decision-making skills.

The results suggest that AI dependence is not negatively associated with critical thinking ability. Recent studies have reported that structured interaction with generative AI may be associated with higher levels of analytical reasoning and reflective learning when students actively engage with AI-generated content (Simoni et al., 2025). From the perspective of CLT, AI tools may reduce extraneous cognitive load by organizing information and providing structured explanations. This reduction may free cognitive resources for germane processing, which could support deeper analysis and schema construction. For nursing interns, who often encounter complex clinical information and time pressure, AI tools may serve as cognitive scaffolds rather than substitutes for reasoning (Labrague et al., 2023). In this context, AI dependence may reflect frequent and task-oriented engagement with AI systems, rather than maladaptive or excessive reliance. Therefore, the observed positive association between AI dependence and critical thinking ability may be understood as reflecting functional engagement with AI tools. However, it is important to interpret this finding with caution. The results do not suggest that increased dependence in a maladaptive sense enhances cognitive performance. Instead, whether reliance on AI supports or undermines critical thinking may depend on how AI tools are used and the extent to which users critically evaluate AI-generated information.

A key contribution of this study is the identification of AI literacy as a potential mediator underlying the association between AI dependence and critical thinking ability. This finding suggests that the association between AI dependence and critical thinking ability may also be related to students’ AI literacy. Previous research has highlighted that the educational benefits of AI depend largely on learners’ ability to assess the accuracy and limitations of AI-generated outputs (El-Banna et al., 2025). This finding can also be understood within the framework of CLT, as AI literacy may help students regulate how cognitive resources are allocated when interacting with AI systems. Students with higher AI literacy are more likely to recognize potential errors, verify information across sources, and reflect on inconsistencies (Rodger et al., 2025). Such behaviors may promote active cognitive engagement and help maintain germane cognitive load, which may in turn support deeper learning. In contrast, low AI literacy may be associated with more superficial acceptance of AI outputs and reduced analytical depth. Thus, AI literacy may be related to cognitive regulation processes and may help explain how AI dependence is associated with critical thinking ability. However, given the cross-sectional design, this pathway should be interpreted as a theoretical explanation consistent with the data rather than as evidence of causality. In addition, these associations should be interpreted with caution, as all variables were measured using self-reported questionnaires at a single time point. Although Harman’s single-factor test suggested that common method bias was not a serious concern, shared method variance may still have partially inflated the observed relationships.

These findings have several practical implications for nursing education. First, rather than restricting AI use, educators should focus on guiding students to use AI critically. Integrating AI literacy training into nursing curricula may help students develop the skills needed to evaluate and apply AI-generated information effectively. Second, clinical instructors may incorporate structured AI-assisted learning activities that encourage verification, reflection, and discussion. By emphasizing critical engagement rather than passive acceptance, AI can be positioned as a cognitive support tool that enhances, rather than replaces, professional reasoning. Despite these limitations, this study provides preliminary evidence that AI dependence and AI literacy are associated with critical thinking ability among nursing interns, offering important implications for the responsible integration of AI in nursing education.

## 5 Limitations and implications

Several limitations should be acknowledged. First, the cross-sectional design limits causal inference. Given the cross-sectional design, these findings should be interpreted as statistical associations rather than evidence of causal relationships or a true mediating mechanism. Second, although AI dependence in this study refers to functional and task-oriented engagement with AI tools, the term may still be interpreted as implying maladaptive or excessive reliance. Third, the participants were recruited from different types of hospitals, more detailed center-level identifiers were not collected. Therefore, potential clustering effects and contextual differences across hospitals, such as training environment, supervision practices, and exposure to AI technologies, could not be fully accounted for in the statistical analysis. Moreover, convenience sampling was used, which may have introduced selection bias and limited the representativeness of the sample. Finally, the study was conducted among nursing interns in a specific educational and cultural context in China. As a result, the findings may not be fully generalizable to other populations, institutions, or healthcare education systems. Future studies should adopt longitudinal or experimental designs, use more diverse and representative samples, incorporate multi-source or objective measures, and apply multilevel approaches to better account for institutional heterogeneity.

## 6 Conclusion

AI dependence is positively associated with critical thinking ability among nursing interns, and AI literacy partially mediates this relationship. The study highlights the importance of promoting AI literacy in nursing education. Rather than limiting AI use, educational strategies should focus on guiding students to use AI tools critically and responsibly. By strengthening AI literacy, nursing programs can help ensure that AI functions as a cognitive support tool that contributes to the development of professional reasoning and clinical judgment.

## Data Availability

The raw data supporting the conclusions of this article will be made available by the authors, without undue reservation.

## Footnotes

1
https://www.wjx.cn/
